# Development of Recombinase Aided Amplification Combined With Disposable Nucleic Acid Test Strip for Rapid Detection of Porcine Circovirus Type 2

**DOI:** 10.3389/fvets.2021.676294

**Published:** 2021-06-25

**Authors:** Wenxian Chen, Jindai Fan, Zhaoyao Li, Yuanyuan Zhang, Yuwei Qin, Keke Wu, Xiaowen Li, Yuwan Li, Shuangqi Fan, Mingqiu Zhao

**Affiliations:** ^1^College of Veterinary Medicine, South China Agricultural University, Guangzhou, China; ^2^Key Laboratory of Zoonosis Prevention and Control of Guangdong Province, Guangzhou, China; ^3^Guangdong Laboratory for Lingnan Modern Agriculture, Guangzhou, China

**Keywords:** porcine circovirus type 2, recombinase aided amplification, disposable nucleic acid detection strip, rapid detection, post-weaning multisystemic wasting syndrome

## Abstract

Porcine circovirus type 2 (PCV2) is the dominant causative agent of PCV2 systemic disease (PCV2-SD) in pigs. It can also associate with other diseases such as respiratory and enteric diseases, reproductive failure, porcine dermatitis and nephropathy syndrome in pigs. Currently, PCV2 infection is a considerable threat in the swine industry. Therefore, it is of great significance to prevent, control, and accurately detect PCV2 in pig farms. Recombinase aided amplification (RAA) technology is an isothermal nucleic acid amplification technology that could rapidly amplify the target gene fragment at a constant temperature. The amplification products labeled with specific molecules could be visually detected using the test strip with the corresponding antibody. In the present study, the RAA technology combined with a nucleic acid test strip (RAA-strip) was established for simple and specific detection of PCV2. Primers and probes targeting the PCV2 ORF2 gene were designed according to the RAA technology principles. The PCV2 RAA-strip established in this study could detect as low as 10^3^ copies/μL of recombinant plasmids containing the PCV2 ORF2 gene fragment. The lowest detection limit about viral DNA and virus titers was 6.7 × 10^−6^ ng/μL and 10 TCID50/mL, respectively. Furthermore, no cross-reaction with other porcine viruses occurred at 37°C and within 15 min. We used 42 clinical samples to assess the performance of our established method. The positive rate of clinical samples detected by PCV2 RAA-strip was 50.00%. This was similar to that detected by PCV2 PCR (45.24%). In conclusion, due to the advantages of strong specificity, high sensitivity, excellent reproducibility, and simple operation method, our PCV2 RAA-strip is suitable for the rapid clinical detection of PCV2 on-site.

## Introduction

Porcine circovirus type 2 (PCV2) is an etiological agent causing the immunosuppressive porcine circovirus disease (PCVD) (1). In the early 1970s, a study discovered a small, spherical cell-like virus particle without pathogenic signs in PK-15 cells and was named as porcine circovirus (PCV) (2). In the 1990s, researchers first found the post-weaning multisystemic wasting syndromes (PMWS) was related to PCVs in Canada and subsequently observed similar symptoms caused by PCVs in pigs in other countries (3–6). By analyzing the difference of genomes and antigens between these two kinds of PCVs mentioned above, scientists confirmed that the PCV causing the PMWs was different from the non-pathogenic PCV in PK-15 cells. Thus, the PCV causing PMWs was called PCV2, and the one without pathological changes in PK-15 cells named PCV1 (7). Subsequently, PCV2 systemic disease (PCV2-SD) was used to replace PMWs to unified and identified the terminology for PCVD. A new circovirus PCV3, considered to associate with porcine dermatitis and nephropathy syndrome (PDNS) (8), was first identified in the United States in 2015 and was subsequently discovered in other countries (9–12). The information of infection characteristic and pathogenesis about PCV3 still unknown and it needs to further identification. In addition to PCV2-SD, PCV2 infection can also cause other diseases like respiratory and enteric symptoms, reproductive failure, and associate with PDNS (13–15). Its genome is 1,700 bp in length and contains two main open reading frames (ORF). ORF1 and ORF2 encode rep protein related to virus replication and cap protein associated with host immune response, respectively (16, 17). PCV2 infection in pigs is widespread globally, and high prevalence has been reported in other areas, including North America, South America, Asia, Australia, Africa, New Zealand, etc. (18). PCV2 can infect pigs of different ages and breeds. Besides, it can infect pigs through horizontal or vertical transmission. This virus can severely destroy the immune system of pigs, hence causing immunosuppression. The low immunity caused by PCV2 infection might lead to the secondary infections of other pathogens, which significantly increased the mortality rate in pigs (19–22). PCV2 has caused substantial economic losses worldwide and severely hampered the development of the pig industry. Due to that PCV2 is an important pathogen that endangers the health of pigs, it is imperative to develop a rapid, sensitive, specific, and accurate on-site diagnostic method for PCV2 detection, which is incredibly critical for disease prevention and control.

With the rapid development of molecular biology techniques, researchers have established several diagnostic methods for pathogens detection. As standard and conventional testing techniques, polymerase chain reaction (PCR) and quantitative PCR (qPCR) techniques have been widely employed by laboratories and firms, because of their rapid, specific, and sensitive characteristics (23–25). However, these approaches require expensive equipment and are inconvenient for field inspection. Due to the advantages of simplicity, rapidity, and high-sensibility, loop-mediated isothermal amplification (LAMP) (26–28) can perform DNA amplification at a constant temperature of 65°C without costly tools. Nevertheless, it possesses a complex primer design program and needs more than 1 h to accomplish nucleic acid amplification. Recombinase aided amplification (RAA) is a recombinase-mediated nucleic acid amplification technology, which predominantly employs three crucial elements including recombinase (obtained from bacteria or fungi), single-stranded DNA binding protein, and DNA polymerase. The recombinase can combine with the primer to form a polymer, then recognize and match the complementary sequence on the template DNA under isothermal conditions. The template DNA is melted with the help of the single-stranded DNA binding protein, and new DNA chains are synthesized under the action of the DNA polymerase. This process is repeated continuously to achieve efficient nucleic acid amplification (29). Besides, RAA amplification products could be easily detected by disposable nucleic acid detection strips, in which the results could be observed rapidly and visually. The probe is added into the RAA reaction system to generate the amplification product labeled with 6-carboxy-fluorescein (FAM) and Biotin, which then could combine with gold-labeled FAM antibody on the sample pad of the disposal nucleic acid test strip. Subsequently, the complex product could flow to the test (T) line through buffer and be captured by Biotin antibody to show a red band. The remaining gold-labeled FAM antibody could be captured by anti-FAM antibody on the control (C) line to produce a red band. In a word, both test (T) and control (C) line could be observed on the strip for positive sample, whereas the negative result only displayed a C line.

In the present study, RAA technology combined with nucleic acid test strips (RAA-strip) targeting the PCV2 ORF2 was developed for PCV2 detection. This method has a great application prospect because of its short detection times, convenience, and practicality of on-site detection.

## Materials and Methods

### Virus and Clinical Samples

PCV2 (YHW strain) was preserved in our laboratory. The genomic DNA or cDNA of swine pathogens including porcine parvovirus (PPV, GD strain), classical swine fever virus (CSFV, shimen strain), Japanese encephalitis virus (JEV, sw/GD/2009 strain), porcine reproductive and respiratory syndrome virus (PRRSV, GD08-2 strain), pseudorabies virus (PRV, Ea strain), and Senecavirus A (SVA, GD-ZYY02-2018 strain) was also stored in our laboratory. DNA of African swine fever virus (ASFV) was a gift from other laboratory. PCV3 ORF2 gene was synthesized and cloned into the pUC57 vector by Tsingke Biological Technology (China). The synthetic plasmid was named as pUC57-PCV3 ORF2. Forty-two clinical tissue samples suspected to be PCV2 infection were collected from pig farms in Hebei province, China.

### DNA Extraction and Plasmid Construction

Viral DNA was extracted using the E.Z.N.A. Viral DNA kit (OMEGA, USA) and stored at −20°C. A pair of PCV2 specific primers (forward primer 5′-GATTAAATTCTCTGAATTGCACA-3′ and reverse primer 5′-GTTACCGGAGCAGAAGACAC-3′) was designed to amplify a 657 bp fragment of PCV2 ORF2 gene. The amplification product was cloned into the pMD19-T vector, and the recombinant plasmid pMD19-T-ORF2 was extracted using E.Z.N.A Plasmid Mini kit I (OMEGA, USA). Plasmid concentration was measured by NANODROP 2000 Spectrophotometer (Thermo Scientific, USA). The recombinant plasmid was diluted from 10^10^ to 10^1^ copies/μL for subsequent sensitivity analysis. The copy number of the recombinant plasmid was calculated according to the following formula:

$$\begin{array}{l} numbers\;of\;copies\;\left(\frac{copies}{\mu L}\right)=\frac{6.02\times 10^{23}\times DNA\;concentration\;\left(\frac{ng}{\mu L}\right)}{length\;\left(bp\right)\times 10^{9}\times 660} \end{array}$$

### PCR for PCV2 Detection

We used conventional PCR detection primers to amplify a 353 bp fragment within the region of the PCV2 ORF2 gene, as previously described (30). A 25 μL volume of PCR amplification reaction consisted of 22 μL of Golden Star T6 Super PCR Mix (Beijing Tsingke Biotechnology Co., Ltd., China), 1 μL of each of the primers (10 μM), and 1 μL of DNA template. The PCR programs were as follows: 98°C for 2 min, then 30 cycles of 98°C for 30 s, 57°C for 30 s, 72°C for 30 s, and a final extension at 72°C for 2 min. Amplicons were tested using 1% agarose gel electrophoresis.

### Design of RAA Primers and Probe

According to the design principle of RAA amplification primers, 3 pairs of RAA primers (Table 1) were designed to amplify the PCV2 ORF2 gene fragment using Primer 5.0 and Oligo 7 software. Then the primers were synthesized by Tsingke Biological Technology (China). The RAA assay was performed in a 50 μL volume using RAA nucleic acid amplification kit (Jiangsu Qitian Gene Biotechnology Co., Ltd., China). In brief, 45.5 μL of the mixture containing 25 μL of Buffer V, 15.7 μL of purified water, 2.4 μL of forward primer (10 μM), and 2.4 μL of reverse primer (10 μM) was added to the reaction tube to dissolve the lyophilized powder. Subsequently, 2 μL of DNA sample and 2.5 μL of magnesium acetate (280 mM) were added to the tubes. Consequently, the reaction tubes were placed into water bath of 37°C to amplify the DNA fragment with 15–20 min. After completion, the amplification products were blended with 1 v/v of chloroform, and the mixture was centrifuged (12,000 rpm) at room temperature for 1 min. Then, the amplification products in the supernatant were detected using a 1.5% agarose gel electrophoresis. In other words, RAA assay combined with agarose electrophoresis analysis was used to screen the optimal primer pair for RAA assay. After obtaining the optimal RAA primer pair, RAA probe was designed to target the amplified fragment by optimal primer pair based on the designing principle of RAA probes. The 5′ end of the optimal reserved primer was labeled with biotin. The probe carried a 6-carboxy-fluorescein (FAM) at the 5′ end and a polymerase extension blocking group (C3 spacer) at the 3′ end, while the 31st nucleotide from the 5′ end was modified with a tetrahydrofuran spacer (THF) (31). All the primers and probes were synthesized by Sangon Biotech (China).

**Table 1:** Primers of recombinase aided amplification (RAA) used in this study.

**Primer name**	**Sequence (5^′^**-3^′^)****	**Position (nt)**
PCV2-RAA-F1	CGGATATTGTATGCCTGGTCGTATTTACTG	1094–1123
PCV2-RAA-R1	TGTAAACTACTCCTCCCGCCATACCATAAC	1312–1283
PCV2-RAA-F2	ACATGGTTACACGGATATTGTATGCCTGGTCGTA	1083–1116
PCV2-RAA-R2	GCCATACCATAACCCAGCCCTTCTCCTACCAC	1295–1264
PCV2-RAA-F3	GTTACACGGATATTGTATGCCTGGTCGTAT	1088–1117
PCV2-RAA-R3	GAGTGGGCTCCAGTGCTGTTATTCTAGATG	1385–1356

### PCV2 RAA-Strip

After obtaining the optimal RAA primers and probe, RAA assay combined with a disposable nucleic acid detection strip (RAA-strip) was established. RAA assay was conducted using RAA nucleic acid amplification kit (Test Strip Method) (Jiangsu Qitian Gene Biotechnology Co., Ltd., China). The RAA reaction system was carried as follows: 25 μL of Buffer V, 15.7 μL of purified water, 2.1 μL of each of forward and reverse primers (10 μM), 0.6 μL of RAA probe (10 μM), 2 μL of DNA template, and 2.5 μL of magnesium acetate (280 mM). The reaction tubes were placed into the water bath of 37°C to amplify the target fragment with 15–20 min. At the end of the reaction, 10 μL of RAA product was dropped into the sample pad of the nucleic acid test strip (Ustar Biotechnologies, China). Then, the nucleic acid test strip was put into the tube containing 100 μL buffer (Ustar Biotechnologies, China) at room temperature and the final result was read within 15–30 min. Both test (T) and control (C) line could be observed on the strip for positive sample, whereas the negative result only displayed a C line. The result was invalid if the C line was not shown on the strip.

### Optimization of Reaction Condition for PCV2 RAA-Strip

In order to obtain optimal reaction temperature for RAA assay, the RAA reaction system was placed in water bath with different temperatures (25–45°C) for 15 min. Then, nucleic acid test strips were used to detect the results. After obtaining the optimal temperature, the RAA assay was performed with different incubation time (5–30 min) using the optimal temperature to verify the optimal incubation time.

### Specificity and Sensitivity of PCV2 RAA-Strip

The specificity of PCV2 RAA-strip was evaluated using different swine-associated viruses including PCV2, PPV, JEV, CSFV, PRRSV, PRV, SVA, and ASFV. pUC57-PCV3 ORF2 was used to assess the specificity of PCV2 RAA-strip.

The sensitivity of the PCV2 RAA-strip method was determined using the recombinant plasmid pMD19-T-ORF2 (10^1^-10^10^ copies/μL, prepared by 10-fold serial dilution), PCV2 DNA (6.7 × 10^−9^ to 6.7 × 10^1^ ng/μL, prepared by 10-fold serial dilution), and PCV2 virus solution (10^−2^ to10^6^ TCID50/mL, prepared by 10-fold serial dilution), which was compared with that of PCR.

### Test of Clinical Samples

Forty-two clinical pig samples from Hebei province were examined for the presence of PCV2 using both RAA-strip and PCR.

## Results

### Establishment of PCV2 RAA-Strip and Optimization of the Reaction Condition

**Figure 1:**
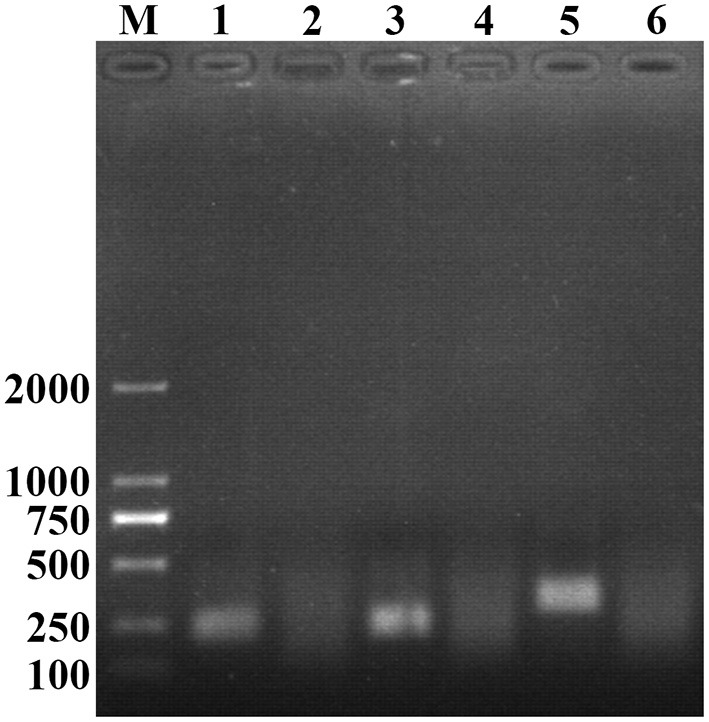
Optimal primer selection. The products amplified by different RAA primers were analyzed by 1.5% agarose gel electrophoresis to select the optimal primer pairs. M is a 2,000 bp DNA marker. The primers and templates used in lane 1–6 successively are: PCV2-RAA-F1/R1+PCV2, PCV2-RAA-F1/R1+ddH_2_O, PCV2-RAA-F2/R2+PCV2, PCV2-RAA-F2/R2+ddH_2_O, PCV2-RAA-F3/R3+PCV2, and PCV2-RAA-F3/R3+ddH_2_O.

Three pairs of primers were confirmed to be specific for PCV2 detection as only one single band with the expected size was observed through agarose gel electrophoresis analysis (Figure 1). According to the brightness of the band and the efficiency of amplification, the third primer pair was selected as the optimal primer pair for RAA assay, and the probe was designed based on the targeted amplification fragment. Detail about the probe and primers was listed in Table 2.

**Table 2:** Optimal primer pairs and probe for recombinase aided amplification (RAA).

**Primer name**	**Sequence (5^′^-3^′^)**	**Position (nt)**
PCV2-RAA-F3	GTTACACGGATATTGTATGCCTGGTCGTAT	1088–1117
PCV2-RAA-B-R3	Biotin-GAGTGGGCTCCAGTGCTGTTATTCTAGATG	1385–1356
PCV2-Probe	FAM-GTTATGGTATGGCGGGAGGAGTAGTTTACA-THF-AGGGGTCATAGGTGA-C3 spacer	1283–1328

**Figure 2:**
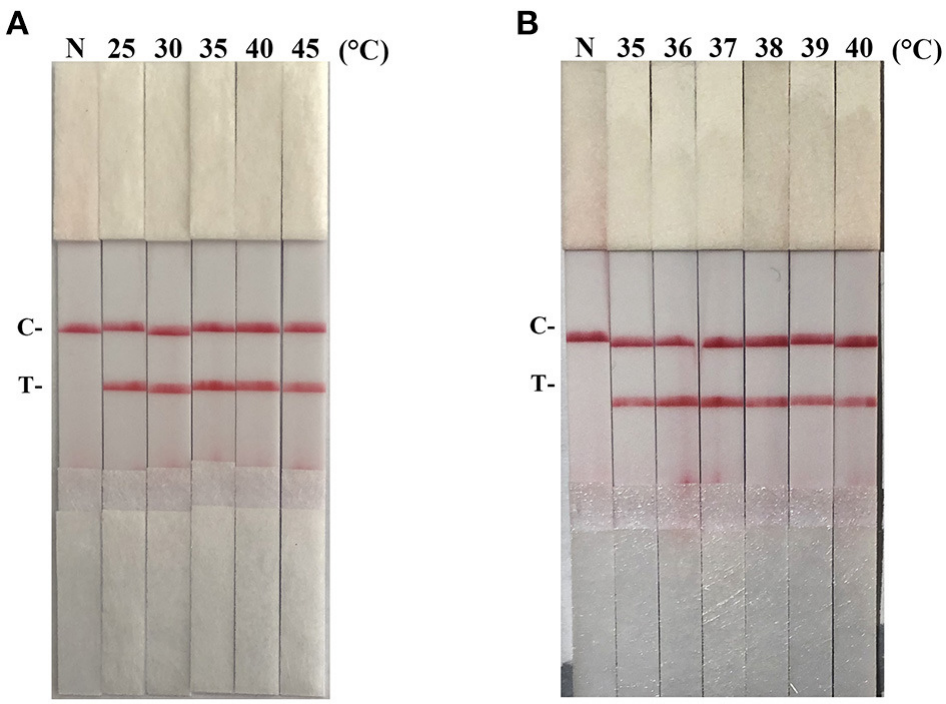
Optimization of reaction temperature. Using the recombinant plasmid pMD19-T-ORF2 as a template to optimize the temperature conditions of PCV2 RAA-strip. C is a control line. T is a test line. N is a negative control. **(A)** The determination of the optimal reaction temperature range of PCV2 RAA-strip. **(B)** The detection of the accurate optimal temperature.

**Figure 3:**
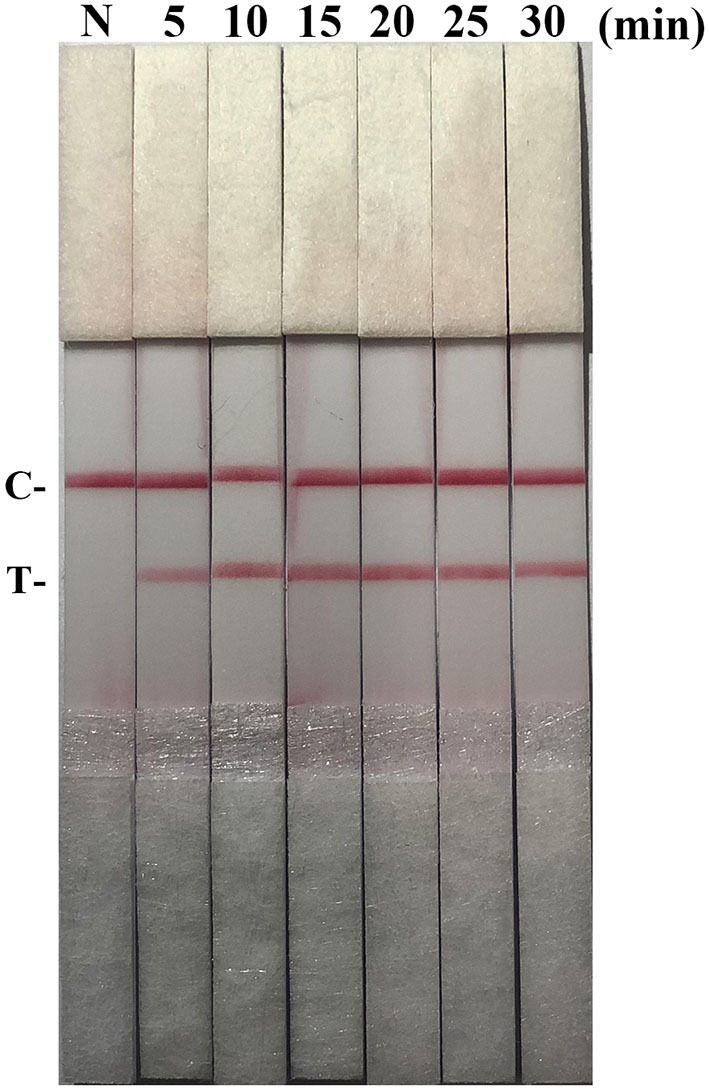
Optimization of reaction time. Using the recombinant plasmid pMD19-T-ORF2 as a template to optimize the amplification time of PCV2 RAA-strip. C is a control line. T is a test line. N is a negative control.

After testing the reaction temperature for RAA assay, we found that the suitable reaction temperature was 35–40°C, and the optimal reaction temperature was 37°C (Figures 2A,B). Then, the incubation time was tested. A weak T line was observed in the strip when the RAA assay was incubated with 5 min. However, the T line appeared clearly on the strip when the RAA incubation time was set to 10–30 min, indicating the rapidity and high-efficiency of this approach. In this study, 15 min was selected as the RAA incubation time (Figure 3).

### Specificity and Sensitivity of PCV2 RAA-Strip

**Figure 4:**
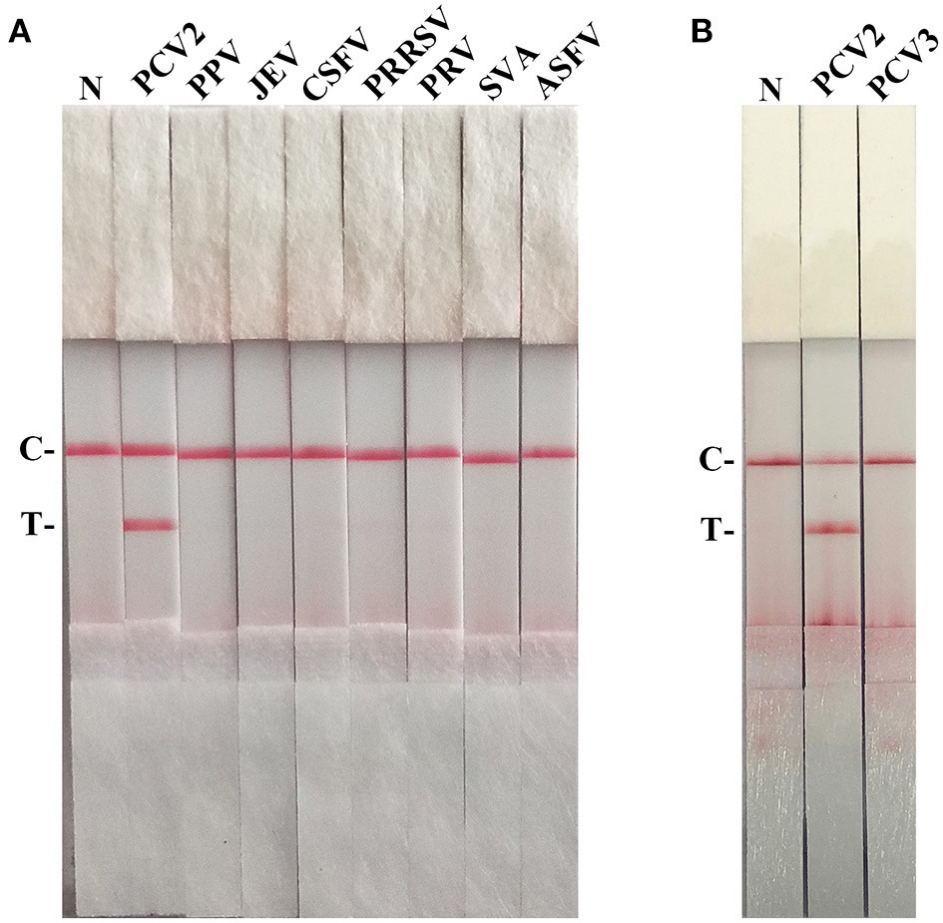
The specificity of PCV2 RAA-strip. C is a control line. T is a test line. N is a negative control. **(A)** The specificity test result of PCV2 RAA-strip using 8 different pig viruses, including Porcine circovirus type 2 (PCV2), Porcine parvovirus (PPV), Japanese encephalitis virus (JEV), Classical swine fever virus (CSFV), Porcine reproductive and respiratory syndrome virus (PRRSV), Pseudorabies virus (PRV), Seneca valley virus (SVV), African swine fever virus (ASFV). **(B)** The specificity test result of PCV2 RAA-strip using PCV2 and Porcine circovirus type 3 (PCV3).

As shown in Figure 4A, the T line appeared for the PCV2-positive sample, and no cross-reaction was observed with other pathogens related to pigs. Besides, PCV2 RAA-strip could not detect PCV3 ORF2 gene (Figure 4B).

**Figure 5:**
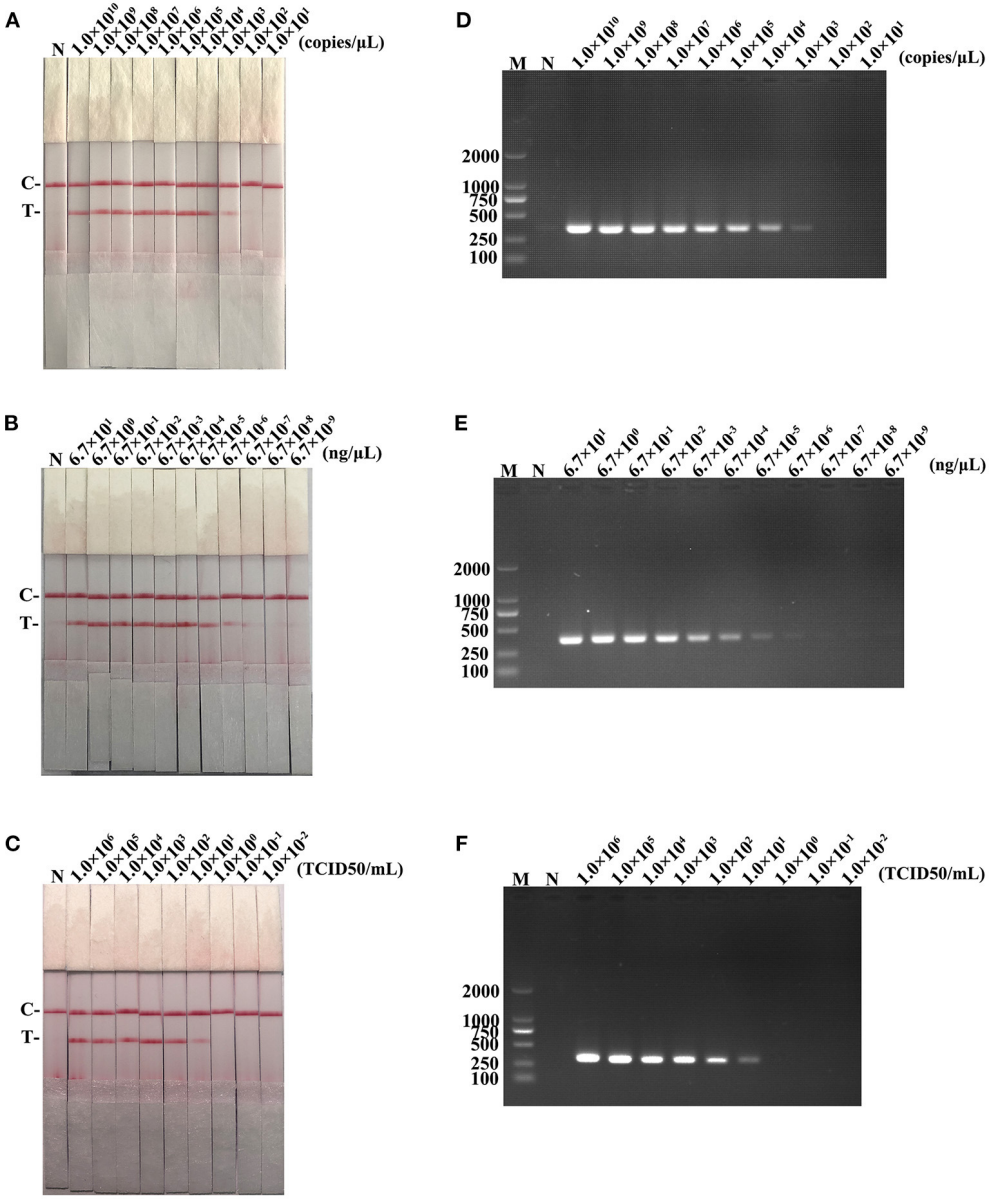
The sensitivity result of PCV2 RAA-strip and conventional PCR. **(A–C)** The sensitivity test result of plasmid copy number, virus DNA concentration and virus titers by PCV2 RAA-strip in order. **(D–F)** Are that by conventional PCR analysis.

The detection limits of recombinant plasmid pMD19-T-ORF2, PCV2 DNA, and PCV2 virus titer were 10^3^ copies/μL, 6.7 × 10^−6^ ng/μL, and 10^1^ TCID50/mL, respectively, for RAA-strip method (Figures 5A–C). In contrast, PCR could detect as low as 10^3^ copies/μL of pMD19-T-ORF2, 6.7 × 10^−5^ ng/μL of PCV2 DNA, and 10^1^ TCID50/mL of virus, respectively (Figures 5D–F). Results showed that the PCV2 RAA-strip has a similar sensitivity with the conventional PCR.

### Clinical Samples Detection

**Figure 6:**
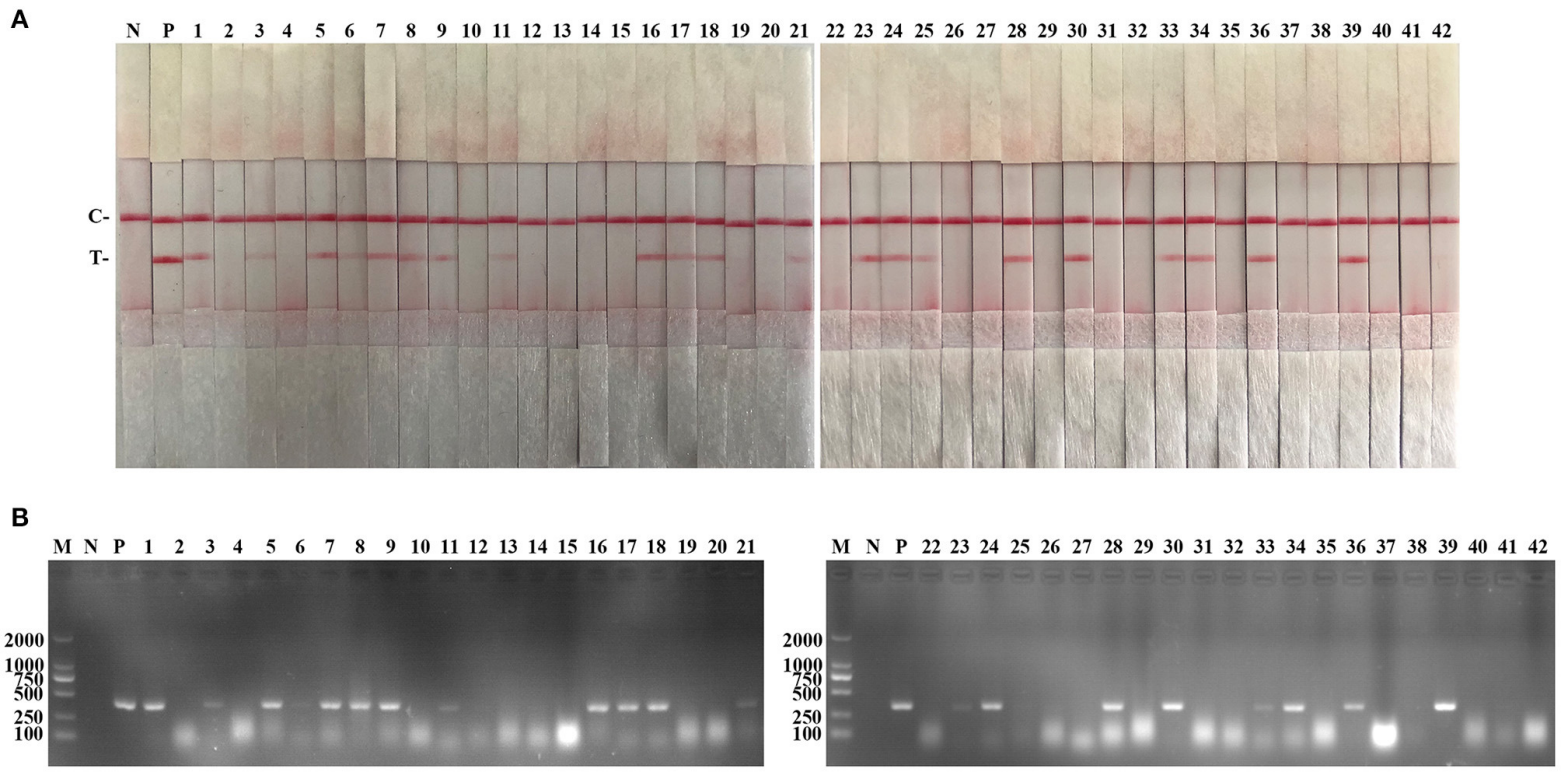
Performance of PCV2 RAA-strip on clinical samples. **(A)** The result of clinical samples detection by PCV2 RAA-strip. **(B)** The result of clinical samples detection by PCV2 conventional PCR. (1–42) are different clinical pig samples reserved in our laboratory. C is a control line. T is a test line. N is a negative control. P is a positive control. M is a 2,000 bp DNA marker.

The performance of the PCV2 RAA-strip was evaluated using 42 clinical samples and compared with that of conventional PCR. As shown in Figure 6 and Table 3, among these samples, 21 samples were tested as positive through PCV2 RAA-strip (50%), whereas the PCR assay confirmed 19 positive samples (45.24%).

**Table 3:** Comparison of PCV2 RAA-strip and conventional PCR in clinical samples.

**Number of samples**	**PCV2 RAA-strip**		**Conventional PCR**	
	**Positive**	**Negative**	**Positive**	**Negative**
42	21	21	19	23

## Discussion

PCV2 is the common pathogen associated with PCV2-SD and immunosuppression in pigs and often triggers secondary infections with other pathogens (32, 33). With the rapid development of the pig industry in China, the scale and intensive degree of livestock breeding has continuously improved under the strong support of the government and society. The government attaches great importance to the prevention and control of swine diseases, and investment in epidemic prevention and control has been increased substantially. The rapid diagnosis and timely control of the disease can effectively prevent disease spread and reduce economic losses. Thus, researchers are committed to develop fast and specific detection techniques for infectious diseases of pigs, which is of great significance for pig diseases prevention.

As a common molecular biology technique, PCR is usually used to amplifying the target DNA fragments while it is time-consuming, and false-positive results can easily occur due to the aerosol contamination in the laboratory. Yang et al. established a detection method of PCV2 using SYBR Green I-based quantitative PCR that could test 10^3^-10^11^ copies of PCV2 DNA per reaction (23). As a result of the high sensitivity characteristics, qPCR has become a useful tool to detect viral nucleic acids in the laboratory. However, it can't work without the aid of precision instruments, and it is unsuitable for on-site test. Liu et al. developed a loop-mediated isothermal amplification (LAMP) test technology for PCV2. This technique detects at least 5.5 × 10^−5^ ng of DNA within 30 min at 63°C (26). Sensitive and specific LAMP has a shorter reaction time and higher detection efficiency than PCR. However, the false-positive outcomes could occur due to the complexity of primer design and the number of primer pairs (34).

RAA technology can amplify the target fragment in 5–20 min under isothermal conditions and requires no precision instruments. After adding labeled probes and primers to the RAA reaction system, samples could be visually detected using test strips. In general, it is suitable for the rapid detection against pathogen infection in the clinical field or poor-equipped laboratories due to the advantages of convenient operation, rapidity, high efficiency and visualization. Both RAA and recombinase polymerase amplification (RPA) are isothermal nucleic acid amplification technologies established in recent years. They have similar principles that can rapidly achieve large-scale nucleic acid amplification under isothermal conditions. The difference between these two methods is the source of recombinase. The recombinase of RPA is uvsX from T4 phage, while the RAA recombinase is extracted from bacteria or fungi, which has the more extensive source (35). RAA is a new technology developed by China that accords with rapid detection demands in our country (36). It has significant advantages, and has been widely used in detecting pathogens (37–41). In this study, we developed the PCV2 RAA-strip which is simple, sensitive, specific for rapid detection of PCV2 within 30 min. Besides, PCV2 RAA-strip is specific for PCV2 ORF2 and no cross-reaction was observed with other pathogens related to pigs and PCV3 ORF2. It is convenient to conduct rapid and direct detection for PCV2 on the spot through PCV2 RAA-strip, which is suitable for remote or poor-equipped areas and has broad application prospects in disease prevention and control. It should be noted that PCV2 RAA-strip needs to be combined with clinical and pathological evaluation of animals to diagnose the disease.

Though less time for detection, simplification of the operation process, and release of the limitations of tools and environment, some improvements are still needed for our RAA-strip. For instance, it is necessary to use viral DNA extraction kits to extract DNA from clinical samples during sample testing. It will be more convenient and faster in clinical testing if the nucleic acid extraction steps can be simplified. Furthermore, according to our sequence analysis, we have confirmed that RAA-strip could detect different subtypes of PCV2. However, there is a need to verify this technique using more PCV2 strains of different subtypes. Additionally, the practicality of RAA-strip needs to be verified using more on-site clinical applications.

## Data Availability Statement

The original contributions presented in the study are included in the article/supplementary material, further inquiries can be directed to the corresponding author/s.

## Author Contributions

WC, SF, and MZ conceived and designed the project. WC, JF, and ZL performed the study and wrote the manuscript. YZ, YQ, KW, XL, and YL participated in preparation of virus and samples. WC, JF, ZL, SF, and MZ revised the manuscript. All authors contributed to the article and approved the submitted version.

## Conflict of Interest

The authors declare that the research was conducted in the absence of any commercial or financial relationships that could be construed as a potential conflict of interest.
